# Supplementary material for: MicroRNA Expression Profiles in Human Samples and Cell Lines Revealed Nine miRNAs Associated with Cisplatin Resistance in High-Grade Serous Ovarian Cancer
Source: Int J Mol Sci. 2024 Mar 28;25(7):3793. doi: 10.3390/ijms25073793 (PMC11011404; doi:10.3390/ijms25073793)
Supplement: Supplementary file 1 [file ijms-25-03793-s001.zip › Supplementary Figures.pptx]

## Slide 1
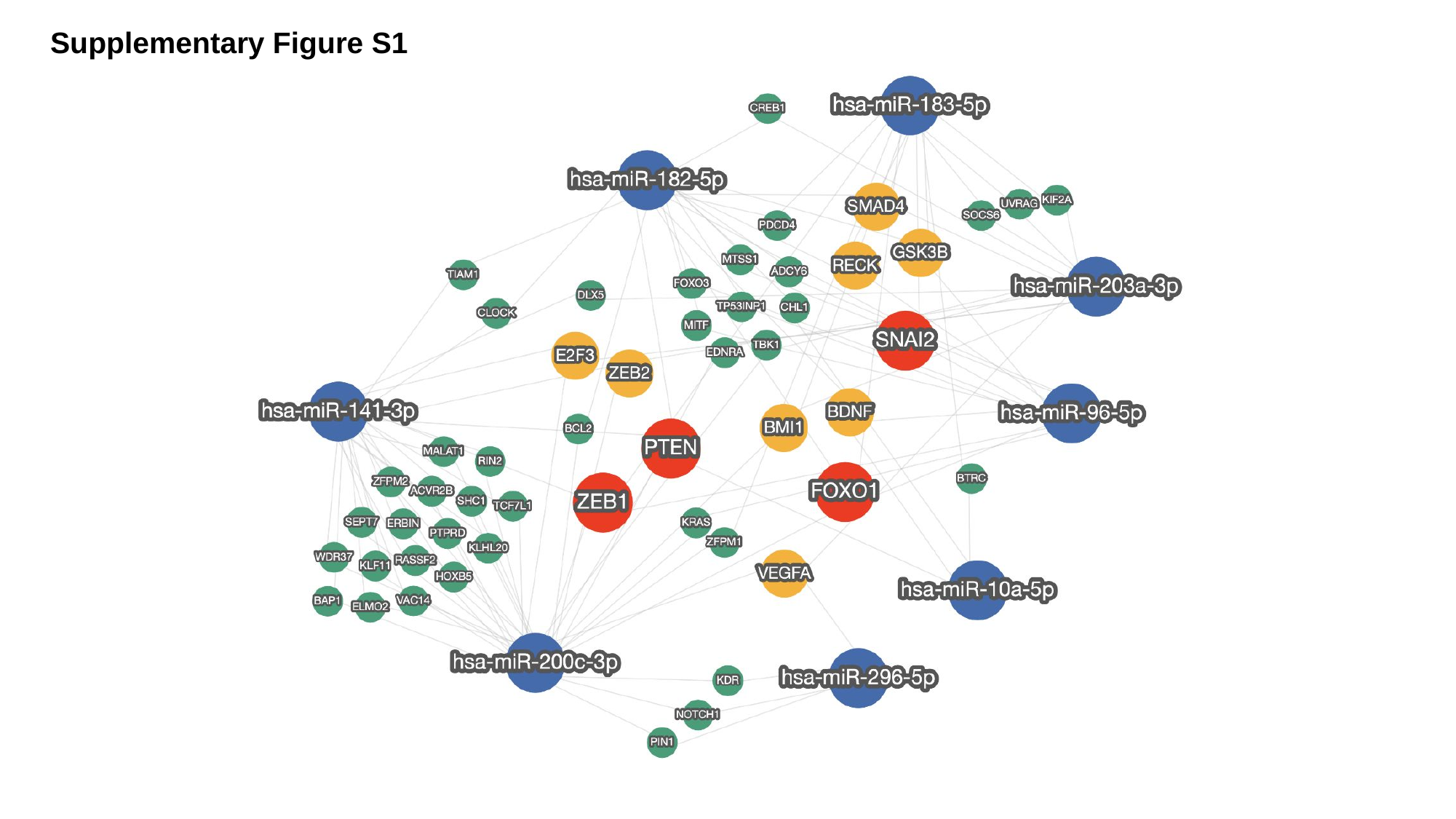

Supplementary Figure S1

## Slide 2
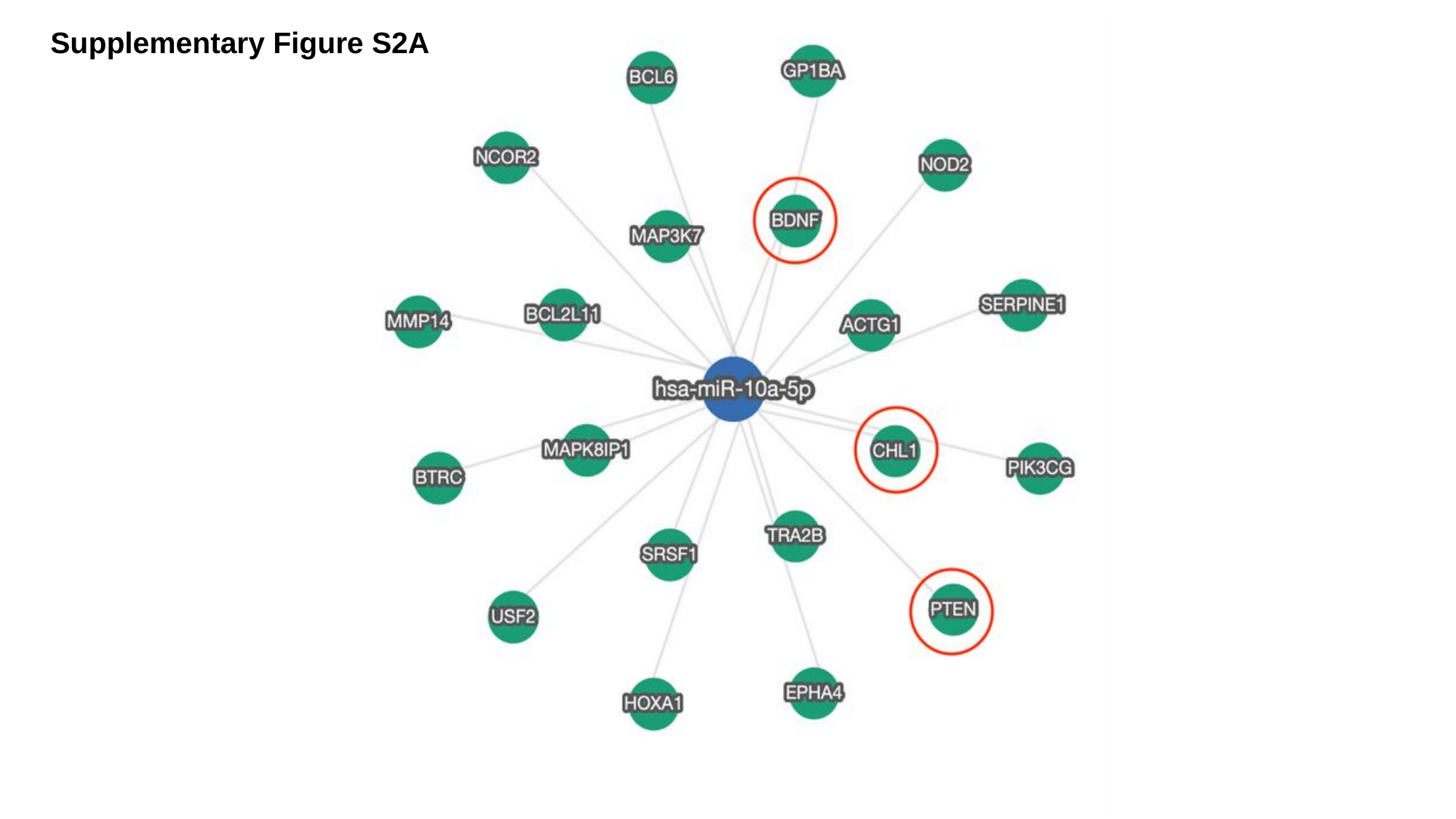

Supplementary Figure S2A

## Slide 3
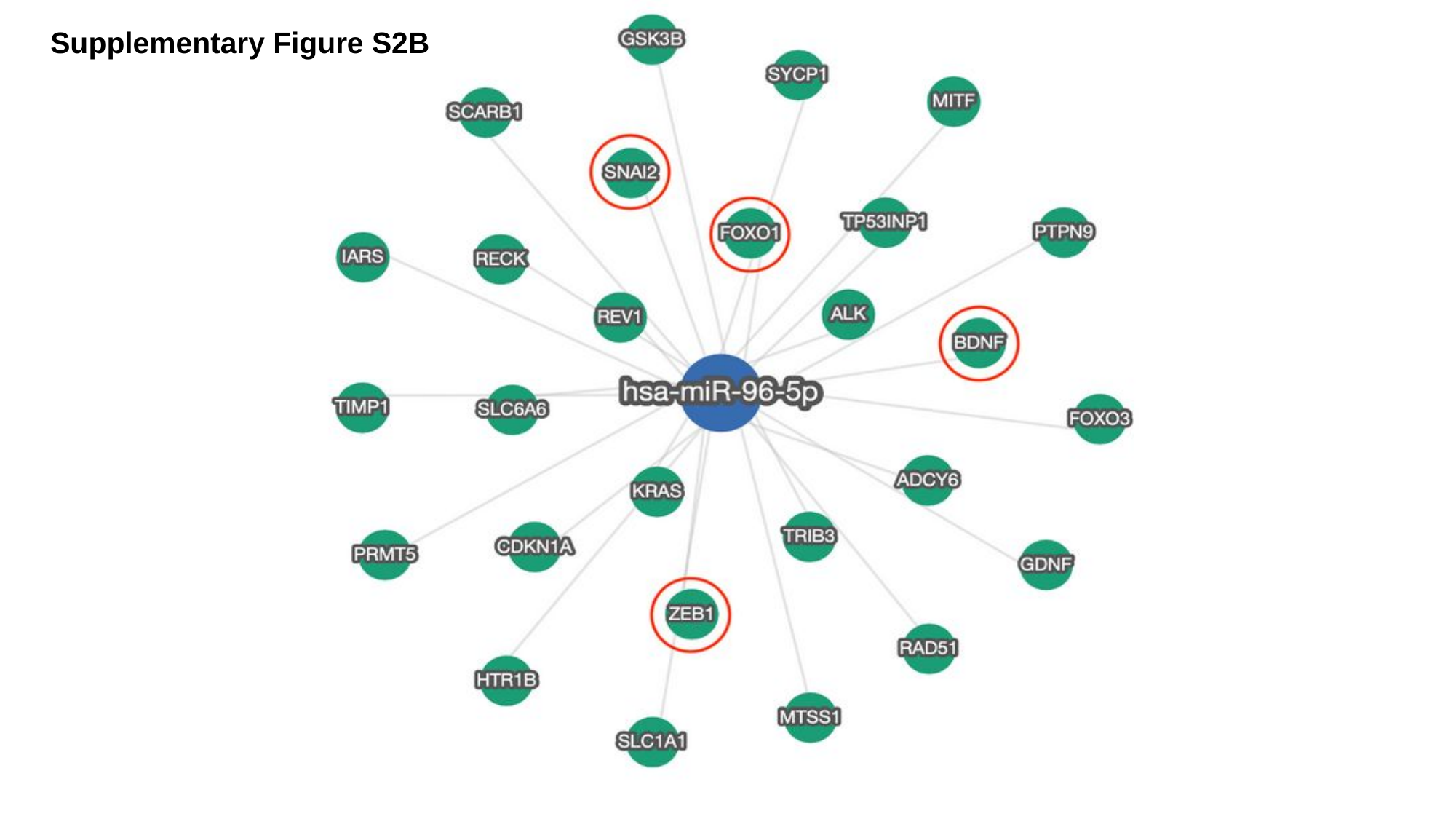

Supplementary Figure S2B

## Slide 4
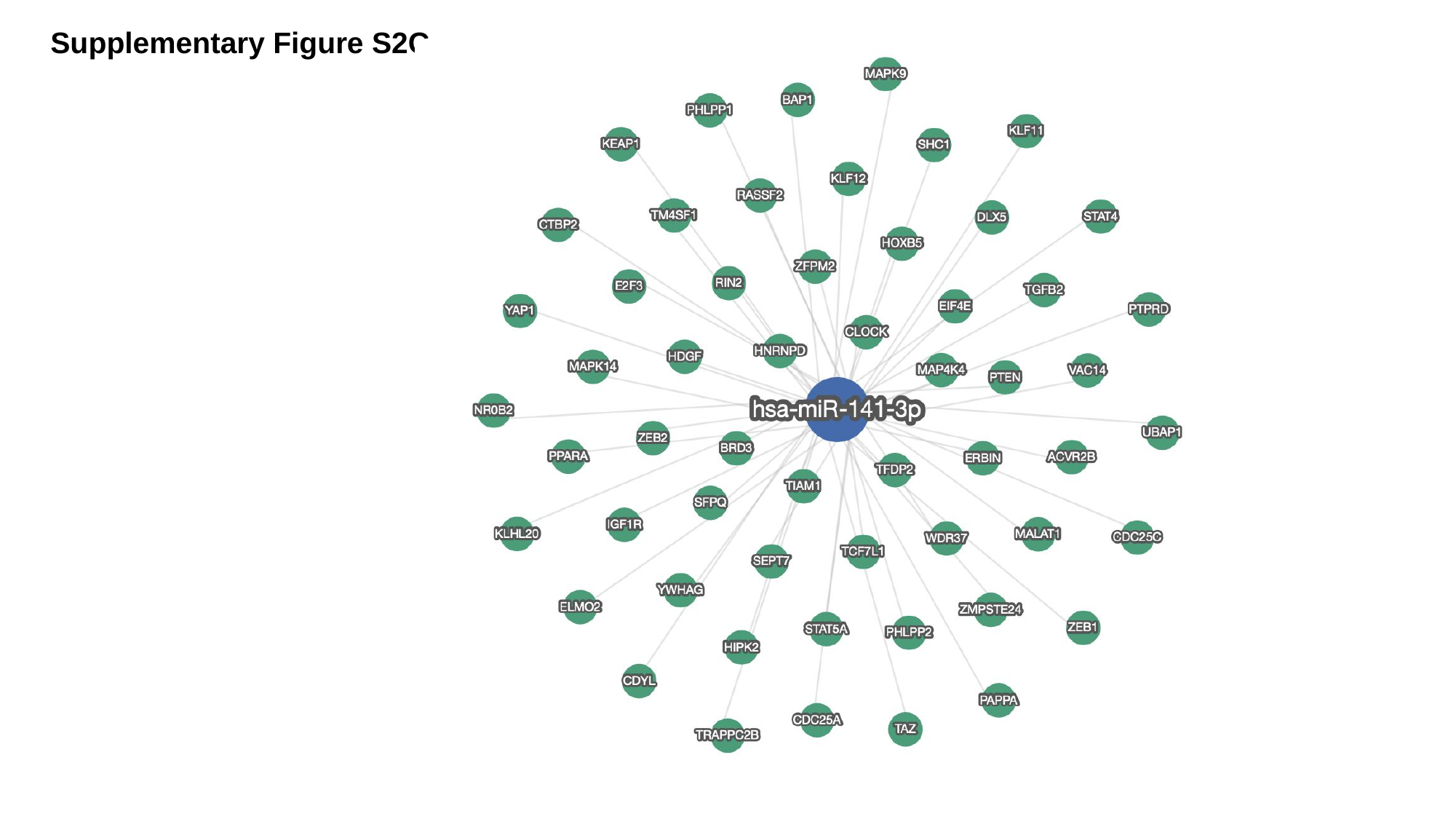

Supplementary Figure S2C

## Slide 5
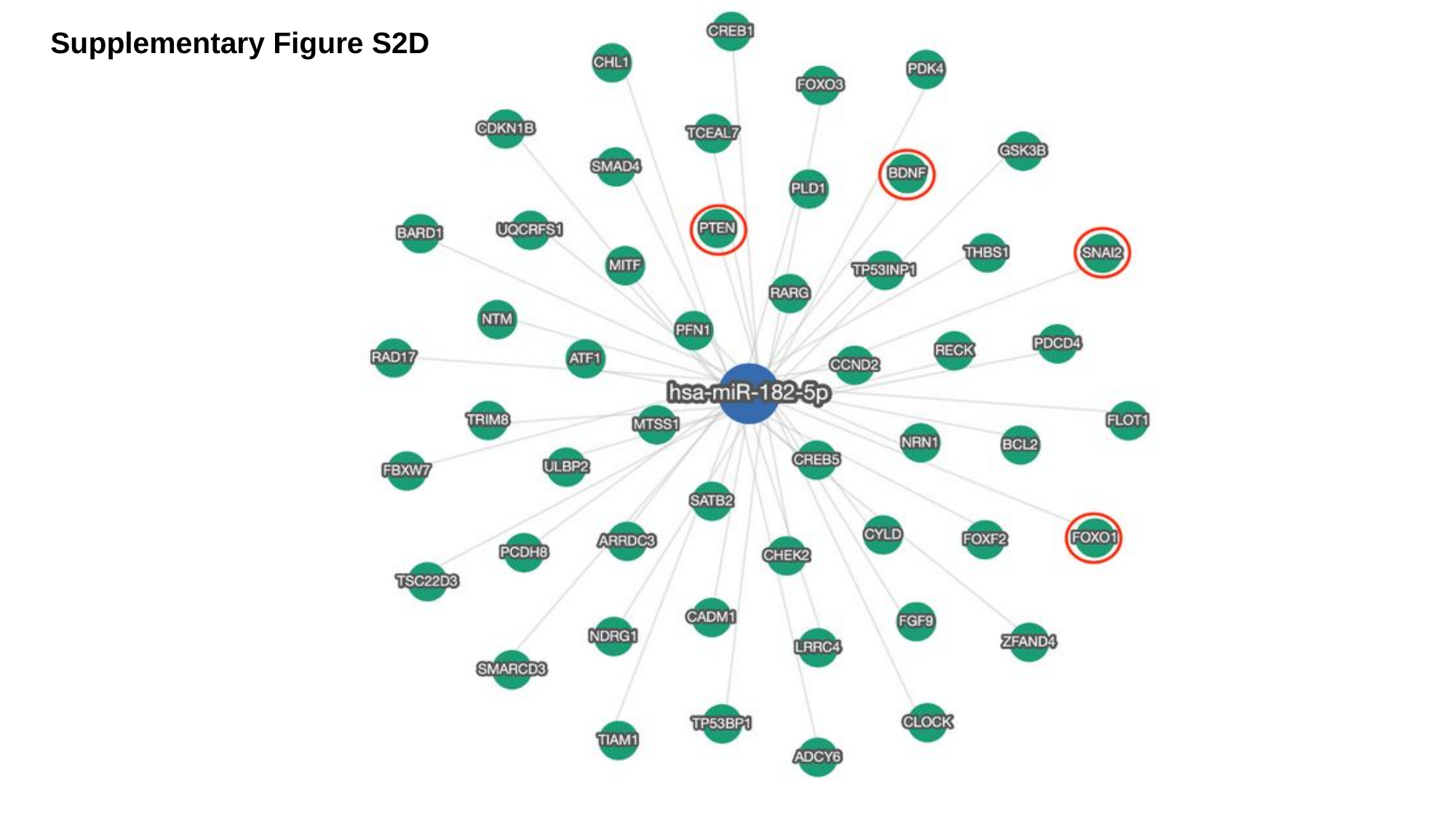

Supplementary Figure S2D

## Slide 6
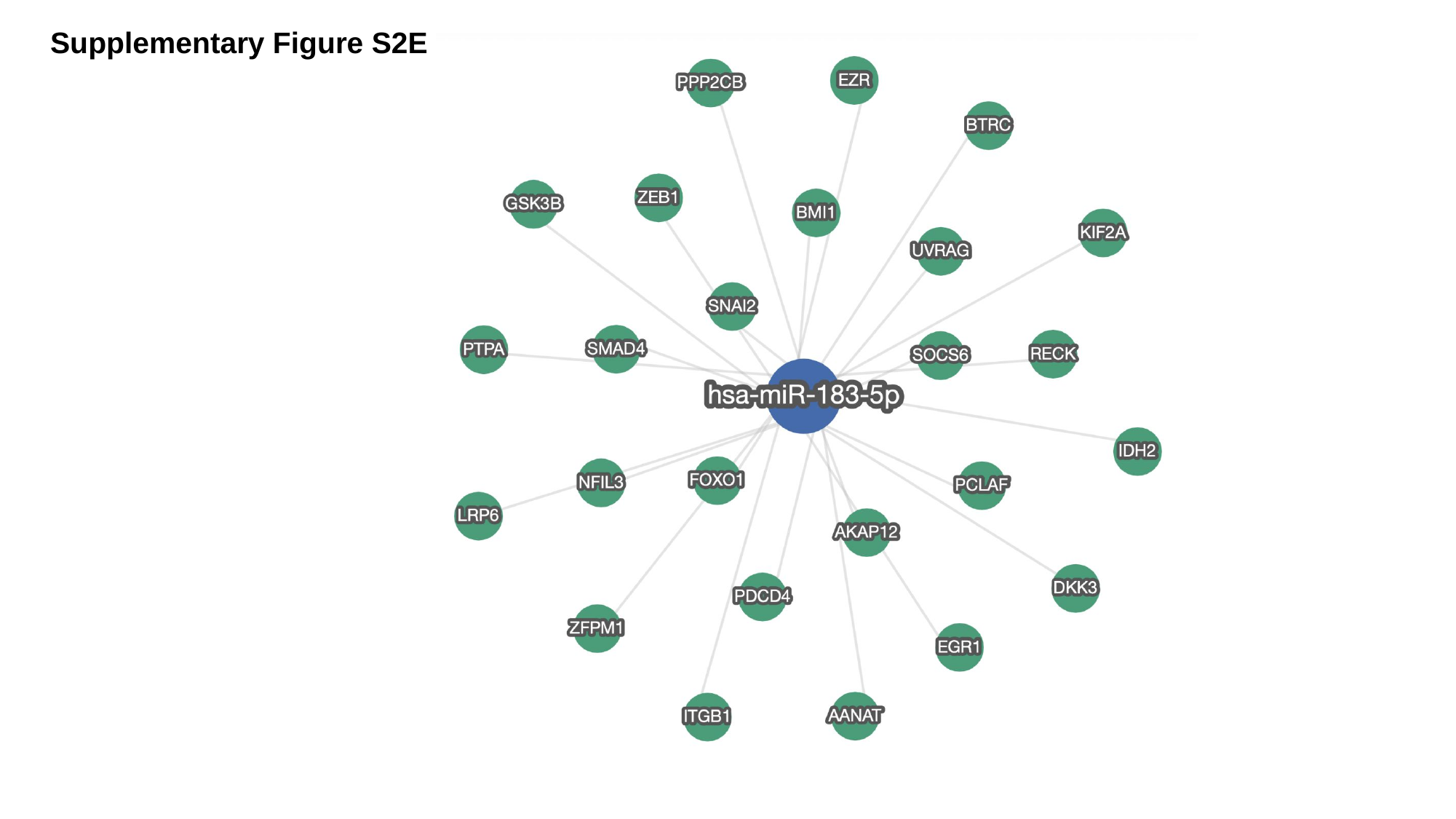

Supplementary Figure S2E

## Slide 7
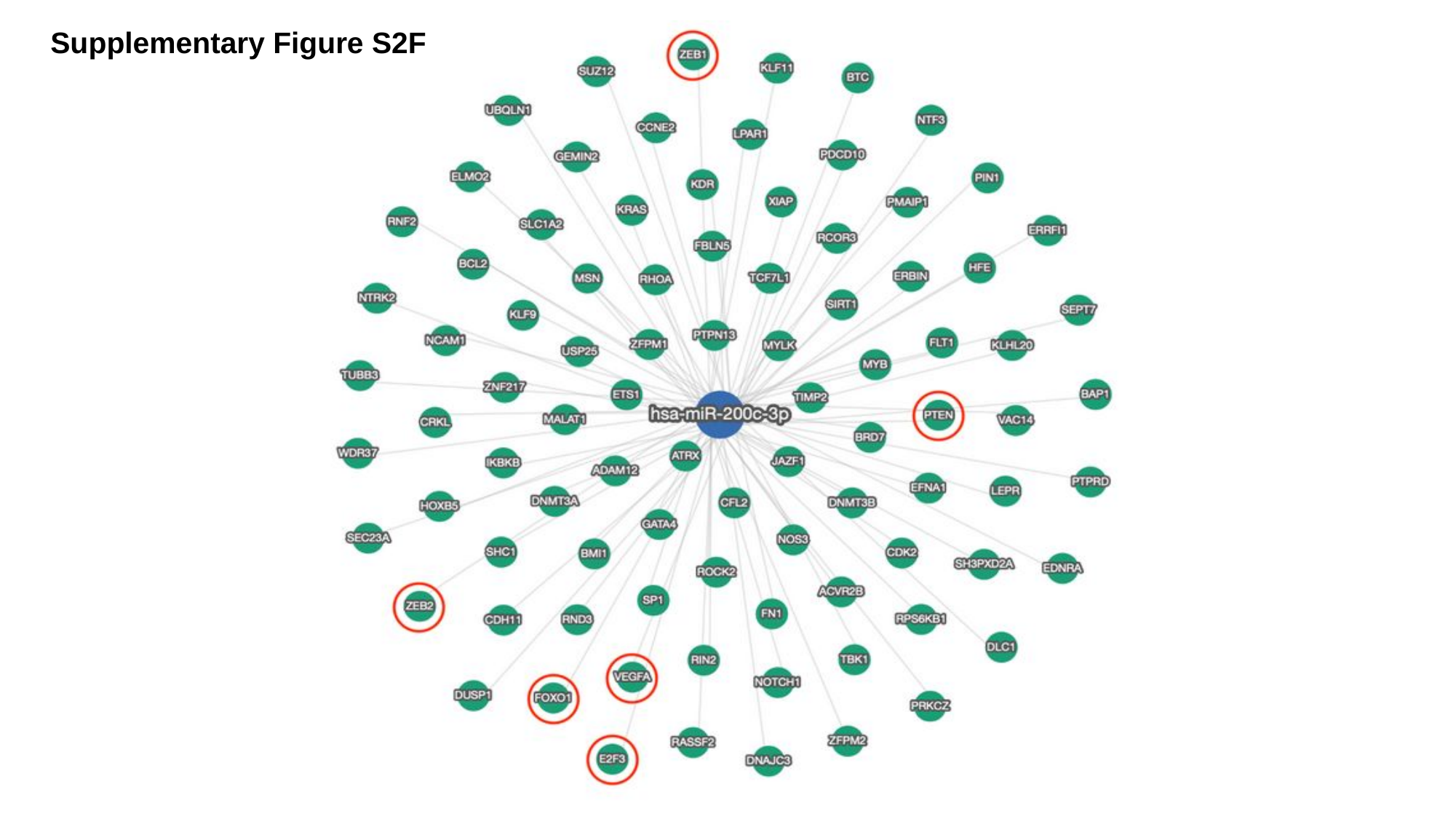

Supplementary Figure S2F

## Slide 8
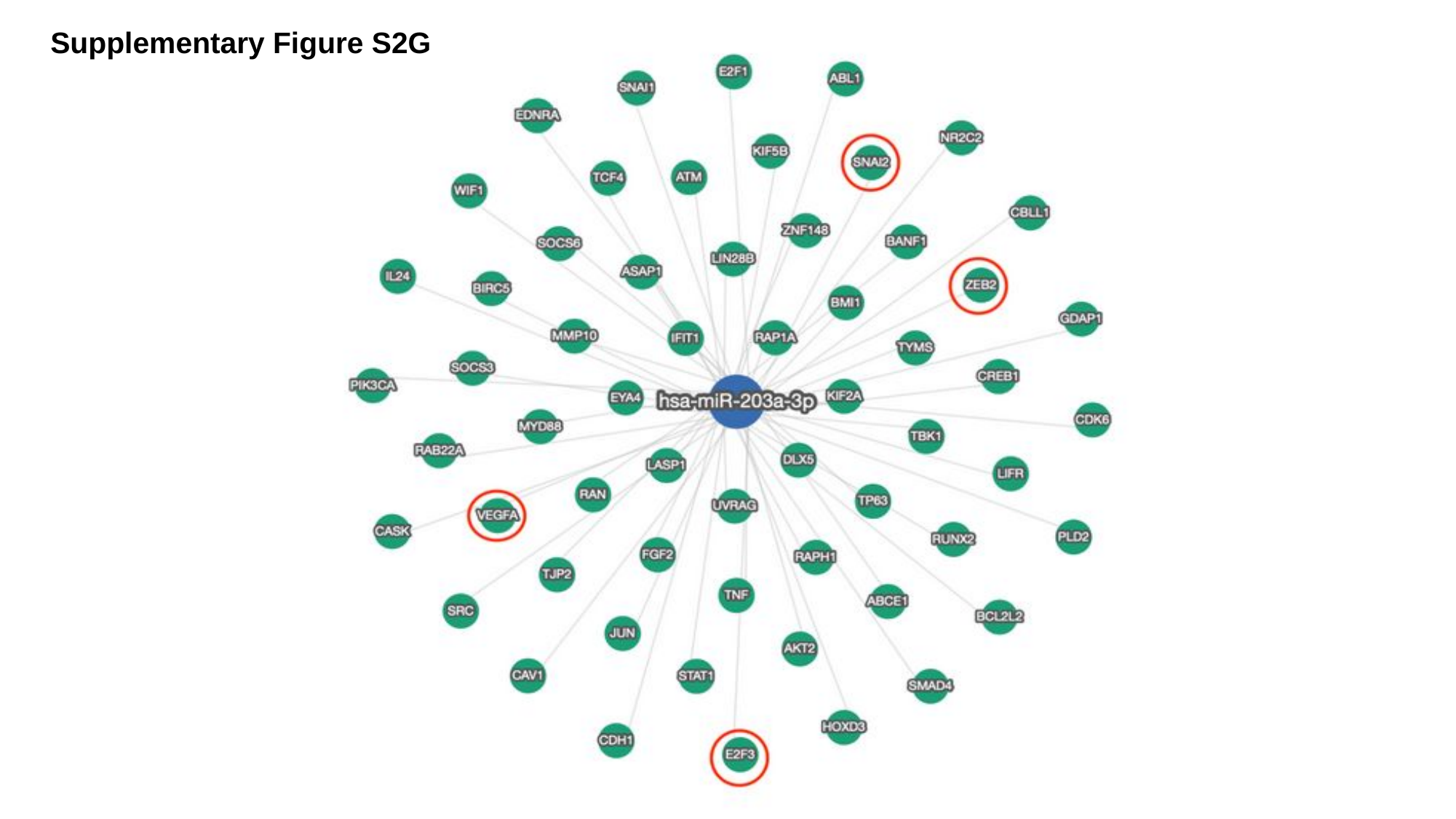

Supplementary Figure S2G

## Slide 9
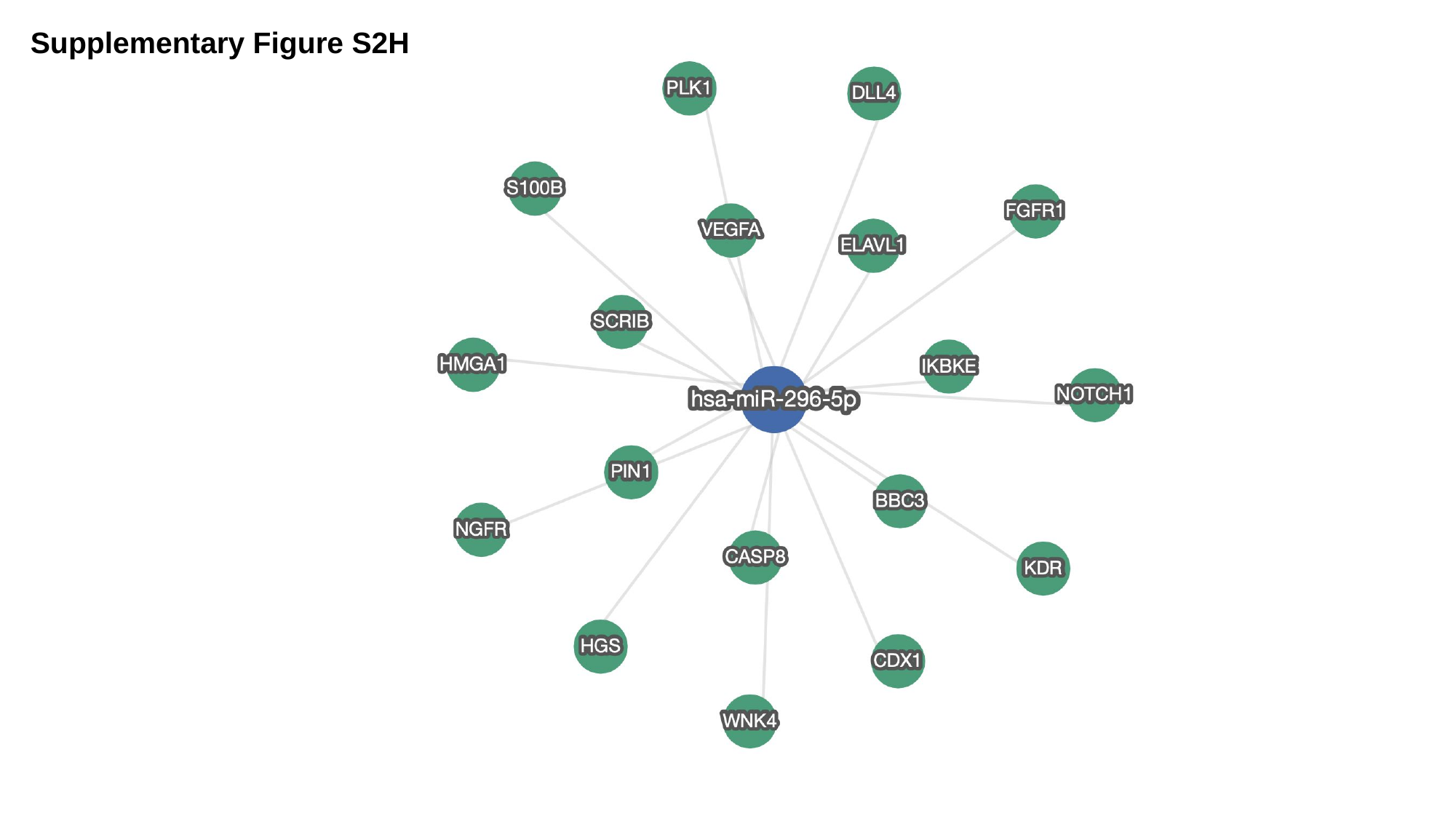

Supplementary Figure S2H

## Slide 10
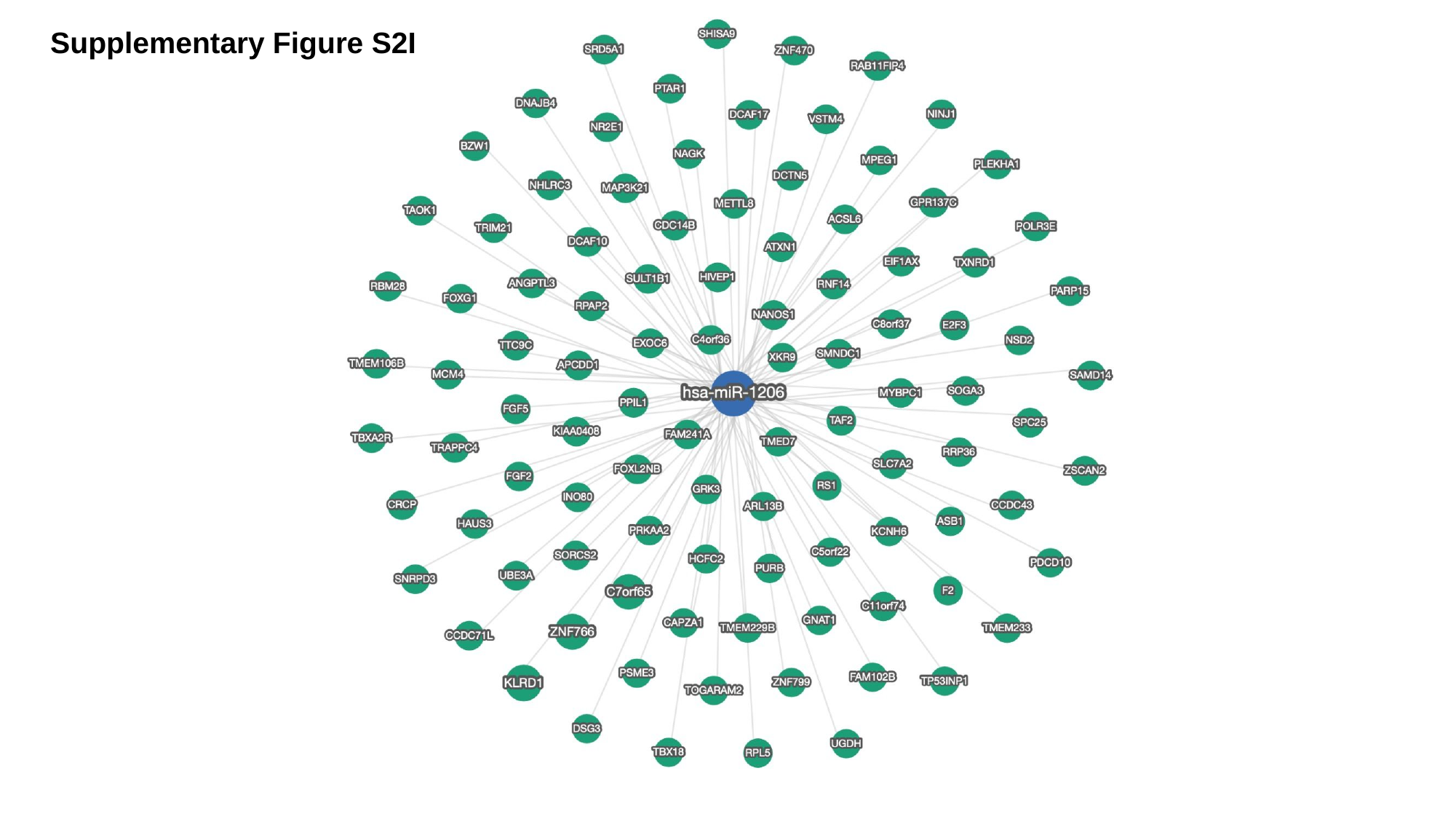

Supplementary Figure S2I
